# What makes orphans in Kigali, Rwanda, non-adherent to antiretroviral therapy? Perspectives of their caregivers

**DOI:** 10.7448/IAS.17.1.19310

**Published:** 2014-12-03

**Authors:** Kimiyo Kikuchi, Krishna C Poudel, John Muganda, Tomoko Sato, Vincent Mutabazi, Ribakare Muhayimpundu, Adolphe Majyambere, Simon P Nyonsenga, Eriko Sase, Masamine Jimba

**Affiliations:** 1 Department of Community and Global Health, Graduate School of Medicine, The University of Tokyo, Tokyo, Japan; 2 Department of Public Health, School of Public Health and Health Sciences, University of Massachusetts-Amherst Amherst, MA, USA; 3 Division of Obstetrics and Gynecology, King Faisal Hospital, Kigali, Rwanda; 4 Department of Psychology, Meiji Gakuin University, Tokyo, Japan; 5 Rwanda Biomedical Center, Kigali, Rwanda

**Keywords:** HIV, AIDS, ART adherence, antiretroviral therapy, orphan, child, Rwanda, sub-Saharan Africa

## Abstract

**Introduction:**

Every year, approximately 260,000 children are infected with HIV in low- and middle-income countries. The timely initiation and high level of maintenance of antiretroviral therapy (ART) are crucial to reducing the suffering of HIV-positive children. We need to develop a better understanding of the background of children's ART non-adherence because it is not well understood. The purpose of this study is to explore the background related to ART non-adherence, specifically in relation to the orphan status of children in Kigali, Rwanda.

**Methods:**

We conducted 19 focus group discussions with a total of 121 caregivers of HIV-positive children in Kigali. The primary data for analysis were verbatim transcripts and socio-demographic data. A content analysis was performed for qualitative data analysis and interpretation.

**Results:**

The study found several contextual factors that influenced non-adherence: among double orphans, there was psychological distance between the caregivers and children, whereas economic burden was the primary issue among paternal orphans. The factors promoting adherence also were unique to each orphan status, such as the positive attitude about disclosing serostatus to the child by double orphans’ caregivers, and feelings of guilt about the child's condition among non-orphaned caregivers.

**Conclusions:**

Knowledge of orphan status is essential to elucidate the factors influencing ART adherence among HIV-positive children. In this qualitative study, we identified the orphan-related contextual factors that influenced ART adherence. Understanding the social context is important in dealing with the challenges to ART adherence among HIV-positive children.

## Introduction

Tremendous efforts have been made to reduce mother-to-child transmission (MTCT) of HIV worldwide. Approximately 260,000 children were infected with HIV in low- and middle-income countries in 2012 [1]. The number is especially high in Sub-Saharan Africa, which accounts for more than 90% of all HIV-positive children in the world [1–3].

To reduce the suffering of HIV-positive children, antiretroviral therapy (ART) should be initiated early and its adherence maintained [1,4–7]. Children in the early developmental stages often need close supervision to take ART properly. However, many children, unaware of their serostatus, often skip taking their medicines, which makes ART adherence in children more complicated than in adults.

Several barriers to ART adherence have been identified among HIV-positive children in Sub-Saharan Africa: only the primary caregiver knows the child's serostatus [8], the child is in conflict with his or her caregivers [9] and the child is an orphan [10–12]. Studies that have examined different orphan status have reported its association with ART adherence [6,8,13,14]. This might be due to the influence of biological relationships on adherence through caregivers’ motivation to implement childcare [13] and the effects of caregivers’ attitudes on children's adherence [8,9]. However, most studies concerning orphan status usually dichotomize the categories as “orphan” and “non-orphan” [6,13].

Rwanda is one of the countries that have taken advantage of ART for HIV-positive children. Of the children needing ART in Rwanda in 2012, 43.0% received it [15]. However, ART adherence remains a challenge, with a rate of 50.5% among children in the capital city of Kigali [16]. In our previous study, double orphans had almost twice the risk of non-adherence as orphans of other statuses, and we identified barriers to and promoters of ART adherence observed in children with different orphan status [16]. Our quantitative results explained limited aspects of adherence, and we needed information on the underlying reasons related to this minority group's social norms [7,17–20], experiences, beliefs and contexts, which are often overlooked [21] but might explain children's motivation and decisions governing ART adherence [17,22,23]. Thus, this study explored the contexts affecting children's ART adherence, according to their orphan status, in Kigali, Rwanda. The question that we posed was “How do different types of orphan status affect ART adherence in these children?”

## Methods

### Setting and study design

We conducted focus group discussions (FGDs) from May to July 2011 with 121 caregivers of HIV-positive children. Kigali city was selected as the study site due to its high number of HIV-positive children and high percentage (23.8%) of Rwanda's paediatric ART services [24]. We used FGDs because it is an optimal method for comprehending participants’ social norms, views and living conditions [17], and it is conducive to stimulating their verbalizations. The study methods and results were reported according to the checklist of consolidated criteria for reporting qualitative research (COREQ) [25] and the relevance, appropriateness and transparency (RATS) guidelines for qualitative research [26].

### Selection of participants

We recruited FGD participants from 717 respondent caregivers who participated in a survey in our previous study on their children's ART adherence [16]. In that study, 717 of the 1301 pairs of caregivers and their children enrolled in an ART programme responded to the survey. They were recruited from 15 health facilities. Four public hospitals and one clinic were selected because they enrolled half of all HIV-positive children in Kigali; and 10 of 21 health centres providing routine paediatric ART services were randomly selected. All of the respondent caregivers were over 18 years of age and the primary caregivers of HIV-positive children. The respondent children were aged six months to 14 years and were enrolled in ART for at least 12 weeks. None of the respondent caregivers or their children had previous relationships with research personnel prior to completing the quantitative survey.

Our interviewers explained the purpose and themes of the FGDs to all of the participant-caregivers in the quantitative survey. To ensure easy communication with the 717 caregivers, those without phone access were excluded, leaving 507 caregivers, of which 365 agreed to participate in the FGDs.

**Figure 1:**
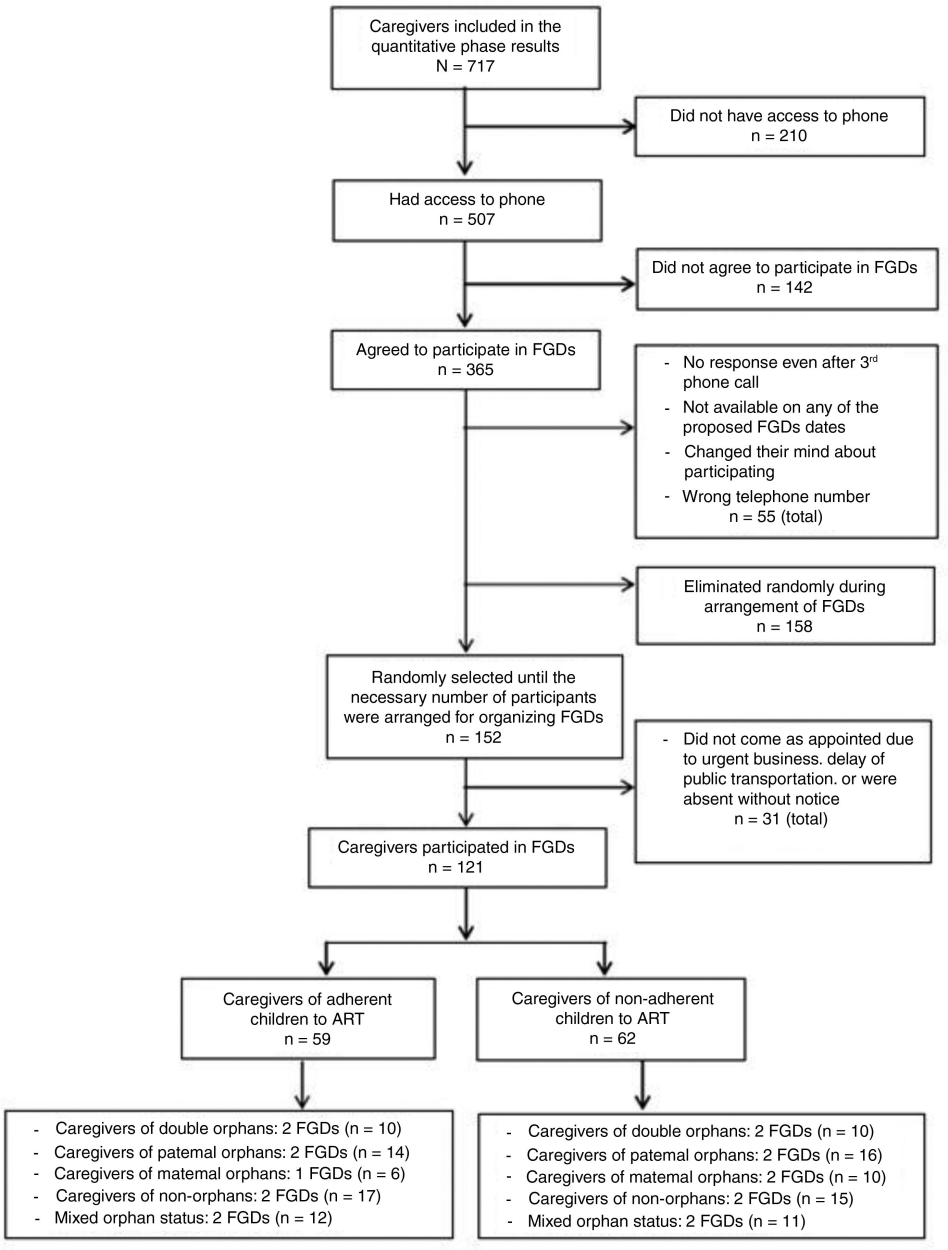
Flowchart summarizing participant inclusion and categories.

The 365 potential FGD participants were classified as adherent or non-adherent, based on the results of our survey. Adherence was measured by pill count, which was previously announced. According to the World Health Organization (WHO) guideline for adherence, a child taking less than 85% of the monthly prescribed pills was considered non-adherent, 85% was poor adherence, and an 85% or higher adherence rate was the minimum level to achieve HIV suppression and the clinical benefits of ART [27]. Caregivers were classified into four categories, corresponding to their child's orphan status: double orphan, paternal orphan, maternal orphan and non-orphan. We arranged two FGDs for each of the eight categories (16 FGDs) with two groups each of adherent and non-adherent caregivers composed of a mix of orphan categories (four FGDs). Two FGDs were organized for each category because two was the minimum number of groups needed to obtain different views of the same category's participants [21]. We could form only one FGD for caregivers of adherent maternal orphans due to the lack of eligible participants (Figure 1).

Of the 365 caregivers, we selected those whom we reached by phone to schedule their FGD date. The caregivers were selected randomly by skipping through the list at regular intervals until the necessary number of participants was recruited. Of the 365 caregivers, 55 were excluded because they could not be reached by phone after three attempts to schedule an appointment (all appointments were made at different times), were unavailable on any of the proposed dates, had changed their minds regarding participation or had an incorrect phone number.

We scheduled FGD dates with 152 randomly selected caregivers from the 310 remaining caregivers. The 158 remaining caregivers were excluded because we had recruited a sufficient number to provide the 8–12 participants needed for each FGD. However, 31 of the 152 selected caregivers did not attend the FGD due to urgent business or public transportation delays, or were absent without notice. Finally, 121 caregivers participated in the FGDs (4 to 10 participants for each FGD). Repeat interviews were not conducted. We held FGDs until we felt that data saturation was achieved.

### Conducting focus group discussions

We conducted the FGDs using a guide that we developed based on a guide previously used in African settings [28]. The topics included barriers to and promoters of adherence, and difficulties perceived by non-biological caregivers. Before conducting the main FGDs, we held three practice sessions with 24 caregivers, and then modified the guide.

The FGDs were conducted in the local language (Kinyarwanda) by a male facilitator (Rwandan medical doctor) with extensive experience in FGDs and a female note-taker (Rwandan nurse) with a sociology background and experience in fieldwork. The note-taker recorded the remarks and reactions of the participants. The discussions were held in a private room inside Kigali's district hospital. Each discussion's duration was 45 to 60 minutes. In addition to the facilitator and note-taker, the first author and her interpreter were present. We used a tape recorder after obtaining participants’ permission. The recordings and the notes were used to produce a complete transcript of each discussion in the local language, which was translated into English for analysis. The transcriptions and translations were produced by different research assistants who had not attended the FGDs. The note-taker of the FGDs reviewed the accuracy of the English translations.

Before each FGD, the facilitator explained the study's purpose, discussion themes and ethical considerations to the participants. Each caregiver provided socio-demographic information and information about the child for whom they cared. Transcripts were not returned to the participants for comments or corrections. However, feedback meetings were held with participants (63 of 121 attended) in November 2011 to obtain their validation and to search for discrepancies in evidence and negative cases.

### Data analysis and processing

The primary data for the analyses were verbatim transcripts and socio-demographic data. The transcripts were analysed by the content analysis method according to the caregivers’ categories (based on the orphan and adherence statuses of the children). We analysed data using the “five-phase cycle,” suggested by Yin [17]: (1) compiling all verbatim transcripts and notes taken, (2) disassembling compiled data using a coding method, (3) reassembling fragmented data by sorting them into different groups, (4) interpreting data by developing data arrays and referring to socio-demographic data and (5) drawing conclusions. Phase (1) was completed by the note-taker, phases (2) and (3) were conducted by the first author and an expert in qualitative research who was not involved in the data collection process, and phases (4) and (5) were performed by the first author. NVivo software, Version 10 (QRS International Pty. Ltd., Doncaster, Australia), was used to sort and code the data. The coding classification scheme was based on the major topical headings specified in the interview guide. Two individuals reviewed and discussed the coded data segments to reach an agreement.

### Ethical considerations

Ethical approval for the study was obtained from the National Ethics Committee of Rwanda and the Research Ethics Committee of the University of Tokyo. Official permission was obtained from the Institute of HIV Disease Prevention and Control/Rwanda Biomedical Centre to collect data at each facility. We started the FGDs after obtaining participants’ written consent and their permission to make digital recordings of the sessions for transcriptions. The discussions were conducted anonymously in a private room, and only a number supplied on the day of the FGD identified the participants.

## Results

Of the 121 caregivers included in the FGDs, 86.8% were women (Table 1). The percentages of caregivers of double orphans, paternal orphans, maternal orphans and non-orphans were 19.0%, 27.3%, 15.7% and 38.0%, respectively. The majority of caregivers were the biological parents of the children. The caregivers’ median age was 36.0 years old, and the children's (42.5% boys and 57.5% girls) median age was 10.0 years old.

**Table 1:** Characteristics of the focus group discussion participants (N=121)

Characteristics	N	(%)
Gender distribution of caregivers		
Male	16	(13.2)
Female	105	(86.8)
Age of caregivers		
Median (IQR) years old	36.0	(11.0)
20–30	27	(22.3)
31–40	54	(44.6)
41–50	31	(25.6)
≥61	9	(7.4)
Gender distribution of children (n = 127)		
Male	54	(42.5)
Female	73	(57.5)
Age of children (n=127)		
Median (IQR) years old	10.0	(6.0)
≤6	36	(28.3)
7–10	35	(27.6)
11–12	27	(21.3)
≥ 13	29	(22.8)
Orphan status of children		
Double orphan	23	(19.0)
Paternal orphan	33	(27.3)
Maternal orphan	19	(15.7)
Non-orphan	46	(38.0)
Relations of caregivers with child		
Parent	83	(68.6)
Sibling	8	(6.6)
Grandparent	2	(1.7)
Uncle/aunt	16	(13.2)
Orphanage staff	3	(2.5)
Other	9	(7.4)
Duration of ART of children (n=127)		
Median (IQR) years	4.0	(3.0)
Children know serostatus		
Yes	63	(52.1)
No	58	(47.9)
Age of children knowing/not knowing serostatus		
Knows serostatus/median (IQR)	12.0	(3.0)
Does not know serostatus	6.0	(3.5)
Professions of caregivers		
Agriculture	8	(6.6)
Manual labourer	13	(10.7)
Small business owner	29	(24.0)
Transportation	3	(2.5)
Student	6	(5.0)
Orphanage staff	2	(1.7)
Temporary job	17	(14.0)
Unemployed	43	(35.5)
Number of children in the household		
0	9	(7.4)
1–2	25	(20.7)
3–4	56	(46.3)
5 or more	31	(25.6)

ART = antiretroviral therapy; IQR = interquartile range.

The overarching discussion theme was the caregivers’ views on how orphan status influenced ART adherence. The factors influencing adherence were classified as caregiver-related factors and child-related factors.

### Caregiver-related factors in adherence

#### Caregivers of double orphans

Although the majority of the caregivers were the biological parents of non-orphans and single orphans, those of double orphans were siblings, aunts, cousins, orphanage staff or neighbours. Caregivers unrelated to the children often expressed a social duty or higher mission as their motivation for childrearing; for example, one said, “It is what God wanted me to do.” Caregivers of double orphans expressed feelings of sympathy or compassion for the “innocent child who is forced to suffer for a disease he/she could do nothing to prevent.”

However, two double orphans’ caregivers felt psychologically distant from their child, which might have been due to their non-biological relationship. These feelings were reflected in the caregivers’ lack of daily support and their impatience with trying to overcome the child's resistance to endless medicine taking. Even the child's siblings often found it difficult to cope with the child:

There are times when a child asks for something that I cannot provide, and the child thinks that I did not give it to him because I am not his parent, and this can make him refuse to take the medicine. (Caregiver of a 12-year-old double orphan, non-adherent)

My brother (an HIV-positive child) usually likes to act kind of lonely, sitting all alone and becoming very unhappy, and when you try to remind him of his medicine, he seems to look away. I start begging him to take it, slowly encouraging him. I think that if I were his mum, he would respond quickly. (Caregiver of a 13-year-old double orphan, adherent)

Among the caregivers of adherent children, willingness and hope were observed in their disclosure of the serostatus to the child. They trusted the child's ability to accept the problem and to take responsibility for confronting the illness by him or herself:

I think after his advanced education, I can tell him everything [about his disease], and encourage him to take the medicine. This is the only way he can survive and he should not get fed up with the medicine. (Caregiver of a 4-year-old double orphan, adherent)

#### Caregivers of maternal orphans

Caregivers of maternal orphans were fathers, maternal or paternal aunts, grandmothers, stepmothers, siblings, cousins or neighbours. Depending on the relationship between caregivers and maternal orphans, the obstacles varied. Two aunts could not be involved in childcare because the child was violent with them. One stepmother mentioned the hindrance of giving the child the medicine in secret and keeping it from the other children. When the father was the only caregiver of the child, he found it difficult to balance his job with childcare:

When you are single, it is not easy to make sure the child takes the medicine. Like when you have to go to work and there is no one to take care of it, when you are not there …. This is an important thing that we would keep secret but you should tell it to at least one person. (Caregiver of a 10-year-old maternal orphan, non-adherent)

#### Caregivers of paternal orphans

Similar to maternal orphans’ caregivers, paternal orphans’ caregivers also expressed difficulties in balancing the child's care with their jobs. They knew that they had “no one to help them to rear their child and to share household duties for fear of the child's serostatus being known.” HIV-positive children need to take medicine at the required time every day. However, when caregivers needed to leave for work early in the morning or stay at work until late at night, they faced problems attending to the child's medication adherence because they could not rely on others:

To get the best things [merchandise] to sell, I have to wake up early in the morning [to go to the market]. I leave the child alone at home. I cannot wake him up, and I cannot tell the neighbours to give him the medicine instead of me. (Caregiver of a 4-year-old paternal orphan, non-adherent)

Lack of food was the main reason for paternal orphans’ non-adherence because the children preferred to take the medicine with something to eat. Nausea or stomach ache often occurred after taking the medicine when nothing was in their stomach. Single mothers who were HIV-positive could not ensure that foodstuffs lasted or would be available at the time the child was scheduled to take the medicine. The mothers often worked temporarily and did not earn a high salary:

My child says, “When I take it [medicine] without anything in my stomach, I feel giddiness, I feel weak and sleepy and vomit …. I do not want to take it.” Only when I am lucky and get something for my child to eat and to drink, that is when the child takes the medicine. (Caregiver of a 14-year-old paternal orphan, non-adherent)

However, caregivers of adherent paternal orphans tried to deal with it: “when you think about how the child used to be and how he is now, you cannot just leave your child without taking medicine, even though there is nothing to take with it.” The caregivers firmly believed in convincing the child to take the medicine even without something to eat by explaining the consequences of non-adherence and the advantages of better adherence.

#### Caregivers of non-orphans

Non-orphan caregivers benefitted from their partner's support when one caregiver was unavailable to help the child take the medicine. Yet, several expressed difficulty in balancing medication administration and their jobs. When the caregiver's spouse did not live with the child because of divorce or separation, the living conditions were similar to those of a single orphan. One stated that he or she mistakenly gave the medicine to the child twice because the child had multiple caregivers:

At 7:15 p.m., I come back home and give the child medicine, not knowing that someone had already given it to him, since young children do not understand; then, you find that the medicine is finished before its refill date. (Caregiver of a 4-year-old non-orphan, non-adherent)

Most of the caregivers of non-orphans were HIV-positive, which positively affected the child's medicine adherence. Usually, the child took the medicine at the same time as the HIV-positive caregiver, which resulted in fewer missed doses:

I take mine [medicine] at the same time as the child to remind him. When I take mine and see that he has not done it yet, I prepare everything. (Caregiver of a 14-year-old non-orphan, adherent)

#### Biological parent caregivers

Biological parents were afraid of incurring their child's enmity and of their child asking them questions about their condition, which might cause other problems. Sometimes they delayed the time of disclosure to the child of his or her condition, which may have promoted, in some cases, the child's adherence to ART. Their feelings of guilt for transmitting HIV to their child might have pushed them to make personal sacrifices for their child's adherence:

It is our fault that the children are sick, so, we need to sacrifice many things so that their lives become easier. You should be a good parent and follow the doctor's prescriptions properly for the children's good. (Caregiver of a 7-year-old paternal orphan, adherent)

### Child-related factors in adherence

#### Developmental stage of the child

Factors influencing adherence differed depending on the child's developmental stage. For very young children, adherence depends on caregivers’ sustained supervision and reminders to take the ART medicine. However, as children age, they begin to reject taking medicine.

#### Emotional conflict

When considering the temperamental nature of adolescents, emotional conflicts with their caregivers also hindered adherence:

When they start to learn everything [about their diagnosis], that is when they feel discouraged and hopeless about their future; that is what I have noticed with my child, he is more difficult to handle as he grows up … when he refuses to take the medicines. It's not because he does not know its importance, but rather it's to make me angry. (Caregiver of a 12-year-old paternal orphan, non-adherent)

#### Stigma

Stigma was a strong factor related to non-adherence. Children who were boarding at school found it difficult to take the medicine without anyone noticing, which led them to skip doses:

When she went to [secondary] school for the first time, she could not take the medicines because there was no way she could be alone. She spent two weeks without taking any and fell ill. She was so ill that she was forced to come back home …. I asked her, “Did you ever take the medicines while you were at school?” She said, “No, I did not. There was no way I could take them. I was always surrounded by people.” (Caregiver of a 12-year-old paternal orphan, non-adherent)

## Discussion

In this study, paediatric ART adherence was closely related to the child's social context, which was derived from their orphan status. Several ART-related barriers seemed to correspond to different orphan statuses, suggesting that each barrier might characterize a particular orphan status. Double orphans’ caregivers often experienced difficulties with providing sufficient support for the child's adherence because they felt psychologically distant from the child. Despite their psychological distance, caregivers of double orphans became involved in the children's treatment, resulting in an increase in their self-confidence and sense of achievement. A poor caregiver-child relationship is a likely barrier to the child's ART adherence, according to a US study [29]. However, the uniqueness of this barrier for double orphans found in the present study was not found in the US study.

The caregivers of paternal orphans experienced ART-related barriers resulting from their poor economic status. Lack of food was identified as a barrier, consistent with the results of qualitative studies on ART adherence in the Democratic Republic of the Congo and Kenya [18,28]. However, the relationship between lack of food and orphan status was not examined. Single orphans’ mothers often suffered from severe poverty. They were employed as temporary workers, earned low wages, reared three or more children alone and faced difficulties keeping the house stocked with food. They often found it difficult, if not impossible, to acquire one daily meal. In those households, routine food shortages caused children to avoid taking ART medicine because it could not be tolerated on an empty stomach. The problem of inadequate food is not limited to HIV-positive children in Rwanda. WHO reported that approximately one-third of the Rwandan population has less than the minimum level of dietary energy consumption [30]. The lack of food has a much greater impact on adherence and physical resistance to disease progression among HIV-positive children than among HIV-negative children. Supporting the food security of HIV-positive children should increase ART adherence, especially for paternal orphans who face food shortages.

While non-biological caregivers were willing to disclose the child's serostatus to him or her, biological parents were afraid of problems arising from the child's knowledge that they had transmitted the virus. In Rwanda, HIV-positive children generally receive excellent psychological care, while the caregivers receive less attention. Caregivers were distressed by disclosing their children's serostatus and depressed by their children's resistance to taking the medicine. A supportive programme for caregivers might prevent their isolation and help them express their anxieties [29].

Children's developmental stage influenced their adherence. When the children were young, adherence depended on caregivers’ constant supervision, which could have been a barrier for single mothers of parental orphans, who might not have sufficient aid. Older children sometimes intentionally skipped doses. They doubted whether they should take the medicine all the time, and preferred the immediate rewards of not taking the medication to the long-term benefits of adherence, similar to the findings reported in a Belgian study [31].

## Limitations

This study has limitations. First, it does not include a sufficient number of male caregivers and caregivers of maternal orphans to reflect their viewpoints accurately. Further research is needed to study why access to male caregivers is limited, especially for maternal orphans. Second, our results might not be generalizable to all HIV-positive children and their caregivers in Kigali because approximately 20% of the participants from the previous study did not agree to participate in this one, we excluded those without phone access, and a substantial number of caregivers dropped out of the FGDs. Finally, it is possible that some participants were telling us what they thought we wanted to hear (social desirability bias).

## Conclusions

Knowledge of orphan status is essential to elucidating the factors influencing ART adherence among HIV-positive children. In our quantitative study, we found an association between orphan status and adherence, but we needed to find what governed this association. Our qualitative study identified specific social-contextual factors influencing non-adherence. Understanding the social context is important for dealing with the challenges to ART adherence among HIV-positive children.
